# How to Build the Plane While Flying: VTE/PE Thromboprophylaxis Clinical Guidelines for COVID-19 Patients

**DOI:** 10.1017/dmp.2020.195

**Published:** 2020-06-16

**Authors:** Alessandro Costa, Eric S. Weinstein, D. Ruby Sahoo, Stanley C. Thompson, Roberto Faccincani, Luca Ragazzoni

**Affiliations:** CRIMEDIM, Research Center in Emergency and Disaster Medicine, Novara, NO, Italy; TEAMHealth Hospitalist Services, Grand Strand Medical Center, Clinical Faculty, Edward Via College of Osteopathic Medicine, HCA Healthcare Journal of Medicine, Grand Strand Medical Center, Myrtle Beach, South Carolina; TEAMHealth Lifepoint Group, Brentwood, Tennessee; Emergency Department, IRCCS San Raffaele, Milano, Italy

**Keywords:** COVID-19, pulmonary embolism, deep vein thrombosis, D-dimer, DOAC

## Abstract

Over the years, the practice of medicine has evolved from authority-based to experience-based to evidence-based with the introduction of the scientific process, clinical trials, and outcomes-based data analysis (Tebala GD. *Int J Med Sci*. 2018;15(12):1397-1405). The time required to perform the necessary randomized controlled trials, a systematic literature review, and meta-analysis of these trials to then create, accept, promulgate, and educate the practicing clinicians to use the evidence-based clinical guidelines is typically measured in years. When the severe acute respiratory syndrome novel coronavirus-2 (SARS-nCoV-2) pandemic commenced in Wuhan, China at the end of 2019, there were few available clinical guidelines to deploy, let alone adapt and adopt to treat the surge of coronavirus disease 2019 (COVID-19) patients. The aim of this study is to first explain how clinical guidelines, on which bedside clinicians have grown accustomed, can be created in the midst of a pandemic, with an evolving scientific understanding of the pathophysiology of the hypercoagulable state. The second is to adapt and adopt current venous thromboembolism diagnostic and treatment guidelines, while relying on the limited available observational reporting of COVID-19 patients to create a comprehensive clinical guideline to treat COVID-19 patients.

The coronavirus disease 2019 (COVID-19) clinical spectrum has presented diagnostic and treatment challenges without evidence-based clinical guidelines (CG). Abnormal laboratory markers of inflammation and coagulation were obtained following prior examples of viral pneumonia evaluation and treatment (severe acute respiratory syndrome, Middle Eastern respiratory syndrome, influenza A virus subtype H1N1). Clinicians noted different laboratory and clinical patterns and began to ask about the hypercoagulable aspects of their patients’ course and publish their observations.

On February 22, 2020, Han et al.^1^ submitted their study concluding, in part, that the D-dimer was found to be predictive of disease progression but did not discuss the clinical implication to recommend therapy. One week later, Lin et al.^2^ published a study concluding that the assessment of the risk of venous thromboembolism (VTE) to prevent the possibility of pulmonary thromboembolism (PE) should include the interpretation of abnormally elevated D-dimer.

The American Society of Hematology (ASH) published a COVID-19 and VTE/Anticoagulation Frequently Asked Question (FAQ)^3^ on March 27, 2020. They recommend continuing the standard pharmacologic prophylaxis and treatment irrespective of COVID-19, because the observed high rates of thrombosis in seriously ill COVID-19 patients in China occurred where routine thromboprophylaxis may not be routinely practiced.

Klok et al. studied 184 COVID-19 pneumonia patients admitted to an intensive care unit (ICU) in the Netherlands from March 7 through April 5, 2020. Despite all patients receiving at least standard VTE prophylaxis doses, 25 (13.5%) had computed tomography angiogram (CTA) proven PE, 3 (1.6%) had a VTE (1 with a proximal deep vein thrombosis [DVT] of the leg and 2 with a catheter related upper extremity thrombosis), and 3 (1.6%) had cerebrovascular thrombotic strokes. The authors proposed that physicians treating COVID-19 pneumonia patients should be vigilant for signs of thrombotic complications, despite standard VTE prophylaxis treatment.^4^

Llitjos et al. studied 26 consecutive patients with all receiving anti-coagulation upon admission, in 2 French ICUs, from 19 March 2020 to 11 April 2020 with severe COVID-19: 8 received prophylactic VTE treatment, with all developing a VTE; and 18 received therapeutic anti-coagulation with 10 developing a VTE and 6 a PE. This study also noted worsening of the PaO2/FiO2 ratio as part of their surveillance while receiving anti-coagulation. The authors concluded to screen for VTE and to implement early therapeutic anticoagulation.^5^

Most clinicians were following the CG of acute respiratory distress syndrome (ARDS) in their COVID-19 pneumonia treatment with the expectation that disseminated intravascular coagulation (DIC), bleeding, would manifest as the spectrum advanced toward multisystem organ failure (MOF). But clinicians raised questions and concluded that patients in the COVID-19 spectrum were hypercoagulable before the DIC and MOF.^6^ They began to treat patients with low molecular weight heparin (LMWH) based on the D-dimer and other readily available laboratory markers acting on what was before them with local study developing treatment plans with little, if any, outcome analysis in part due to lack of autopsy, imaging, and other definitive measures.^7-10^

The letter to the editor by Barrett et al.^11^ responded to the International Society on Thrombosis and Hemostasis (ISTH) interim guidance on recognition and management of coagulopathy in COVID-19 from 22 March 2020 that recommended only prophylactic VTE treatment.^12^ They provided a logical argument that, due to the hypercoagulable state, coagulation management needs to be pushed to the forefront.

Available VTE/PE prophylaxis guidelines^7-10^ have been collected with various dates of inception based on their own institutional or network data as well as early publications regarding the hypercoagulable state to assist this study. How to build CG (the plane) based on the findings of a scoping review while practicing (flying) in the early phase of the COVID-19 pandemic uses an understanding of CG development as the science becomes refined while carefully examining measurable outcomes of the incidence of VTE and PE when available. All-cause mortality^13^ without autopsy or confirming imaging of PE, complications from the LMWH treatment, and control for adherence to the adopted local CG with set D-dimer parameters based on prophylaxis and treatment LMWH dosages can be followed by the institutions that adapt, adopt, and create their own CG.^14,15^ The aim of this scoping review is to determine the options for anti-coagulation therapy during the SARS-nCoV-2 clinical spectrum from available peer-reviewed literature from clinical researchers’ observations, some published on-line ahead of print during the study. Specifically of interest is to learn the change in the D-dimer over a period of time that would indicate when to escalate the LMWH dose from standard VTE/PE prophylaxis to VTE/PE treatment.

## METHODOLOGY

A scoping review is preferred over a systematic review in a pandemic, as it is able to quickly identify the evidence available to identify knowledge gaps, scope a body of literature, and clarify concepts.^16^ During the SARS-n-CoV-2 pandemic, an increase in scientific production has occurred, with on-line publication ahead of print, providing a massive amount of data in a span of weeks. As many different studies, with different sizes and search questions, were hastily published to spread knowledge on the COVID infection, scoping reviews are specially fitted to collect the essence from different studies and highlight emerging evidence in current peer- reviewed literature when it is still unclear what other, more specific, questions can be posed and valuably addressed by a more precise systematic literature review.^17^

## METHODS

### Search Questions

Which D-dimer levels require clinical actions in COVID-19 patients?

What COVID-19 hypercoagulation evaluation and treatment data are available in the midst of the pandemic?

How common are thrombotic and hemorrhagic events in the COVID-19 spectrum?

### Information Sources

PubMed electronic records were searched for publications regarding, coronavirus and anticoagulation, using the key words *“coronavirus” or “COVID” or “SARS-nCov-2” AND* the search terms listed in Table 1.

**TABLE 1:** Search Keywords

Key Words	Yielded Results
“d-dimer”	49 results
“anti-coagulation” or “anticoagulation” or “thrombotic complications” or “anticoagulant treatment” or “hemostasis” or “hemostasis” or “anticoagulant treatment”	50 results
“NOAC” or “DOAC” or “LMWH” or “enoxaparin” or “dabigatran” or “rivaroxaban” or “apixaban” or “edoxaban” or “heparin” or “warfarin” or “Coumadin”	16 results
“Pulmonary embolism” or “DVT” or “deep vein thrombosis” or “VTE” or “venous thromboembolism”	15 results

Search keywords used with the results for each query. The strings reported in the table were alternatively put in in the parenthesis of the search query: “coronavirus” or “COVID” or “SARS-nCoV-2” AND (*).

### Eligibility Criteria and Selection Process

Papers that met inclusion criteria were exported from PubMed as XML files and entered on a Google spreadsheet, which included the following fields: Subject title, Authors, Name of the journal or book, Abstract, Date of publication.

Inclusion: Studies published from 1 January to 23 April 2020, English studies, Chinese studies with an English abstract.

Exclusion: Studies that did not answer the search questions, Single case reports, Papers that reported data from other studies.

The first screening was a review of the title, by 2 independent reviewers (A.C., E.W.) each reviewer would note, “yes” or “no” on the spreadsheet. If there was “yes” consensus, the article was included in the second screening review. If there was “no” consensus, the article was discarded. If consensus was not reached, the 2 reviewers discussed the subject, and then, if consensus was not reached, then the third reviewer (D.S.) was the arbiter.

The second screening was a review of the abstract, again noting, “yes” or “no” on the spreadsheet. If there was “yes” consensus, the article was included in the scoping review. If there was “no” consensus, the article was discarded. If consensus was not reached, the 2 reviewers discussed the subject, and then, if consensus was not reached, then the third reviewer (D.S.) was the arbiter.

Following the screening process, data were entered on to the spreadsheet for the following elements: D-dimer levels of COVID patients, thrombotic and hemorrhagic events of COVID patients, protocols and therapeutic approaches for the use of anticoagulants in COVID patients, open questions and unanswered research questions on coagulation and related complications, D-dimer values that trigger the start of anticoagulation, first anticoagulation drug used and relative dosing, D-dimer values that trigger the escalation of anticoagulation, surveillance subjective (symptoms) or objective (labs, imaging) data to monitor complications of the hypercoagulable state or treatment, escalation of anticoagulant therapy, discharge with anticoagulant therapy.

## RESULTS

**FIGURE 1:**
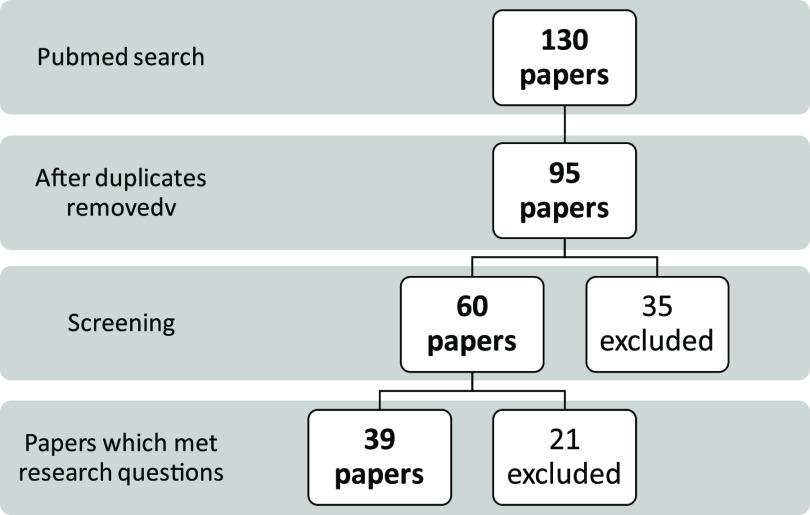
Scoping Review

The search retrieved 130 publications, once 35 duplicate results were removed, 95 papers were screened then 35 were excluded.^1,2,4,5,9,10,13,18-47^ Based on title and abstract after the second screening, 60 papers were selected for further review. There were 5 Chinese papers with an English abstract and Chinese-only full text that made it to the second phase, and it was agreed by both reviewers to evaluate these based on the abstract (Figure 1).

The expanded Google spreadsheet served as a data extraction tool for the 39 papers that were selected to have met the search questions (Table 2).

**TABLE 2:** Search Results

Title	Authors	Journal/Book	D-Dimer	DVT/PE	Therapy
[The keypoints in treatment of the critical coronavirus disease 2019 patient]	Li XY, et al.	Chinese Journal of Tuberculosis and Respiratory Diseases	x	x	
Analysis of clinical characteristics and laboratory findings of 95 cases of 2019 novel coronavirus pneumonia in Wuhan, China: a retrospective analysis	Zhang G, et al.	Respiratory Research	x		
Clinical and immunologic features in severe and moderate Coronavirus Disease 2019	Chen G, et al.	J Clin Invest	x		
Clinical characteristics of 113 deceased patients with coronavirus disease 2019: retrospective study	Chen T, et al.	BMJ (Clinical research ed.)	x		
Clinical characteristics of 140 patients infected with SARS-CoV-2 in Wuhan, China	Zhang JJ, et al.	Allergy	x		
Clinical characteristics of 25 death cases with COVID-19: a retrospective review of medical records in a single medical center, Wuhan, China	Li X, et al.	Int J Infect Dis	x		
Clinical course and risk factors for mortality of adult inpatients with COVID-19 in Wuhan, China: a retrospective cohort study	Zhou F, et al.	The Lancet	x		
Clinical features and treatment of COVID-19 patients in northeast Chongqing	Wan S, et al.	J Med Virol	x		
Diagnostic utility of clinical laboratory data determinations for patients with the severe COVID-19	Gao Y, et al.	J Med Virol	x		
Elevated Plasmin(ogen) as a Common Risk Factor for COVID-19 Susceptibility	Ji HL, et al.	Physiological Reviews	x	x	
Prevalence of venous thromboembolism in patients with severe novel coronavirus pneumonia	Cui S, et al.	J Thromb Haemost	x	x	x
Prominent changes in blood coagulation of patients with SARS-CoV-2 infection	Han H, et al.	Clin Chem Lab Med	x		
Risk Factors Associated With Acute Respiratory Distress Syndrome and Death in Patients With Coronavirus Disease 2019 Pneumonia in Wuhan, China	Wu C, et al.	JAMA Intern Med	x		
D-dimer is Associated with Severity of Coronavirus Disease 2019: A Pooled Analysis	Lippi G, Favaloro EJ.	Thromb Haemost	x		
Abnormal coagulation parameters are associated with poor prognosis in patients with novel coronavirus pneumonia	Tang N, Li D, et al.	Journal of thrombosis and hemostasis: JTH	x		
Thromboprophylaxis and laboratory monitoring for in-hospital patients with COVID-19 - a Swiss consensus statement by the Working Party Hemostasis	Casini A, et al.	Swiss Med Wkly			x
Hypothesis for potential pathogenesis of SARS-CoV-2 infection-a review of immune changes in patients with viral pneumonia	Lin L, et al.	Emerging microbes & infections			x
Anticoagulant treatment is associated with decreased mortality in severe coronavirus disease 2019 patients with coagulopathy	Tang N, Bai H, et al.	J Thromb Haemost	x		x
Difference of coagulation features between severe pneumonia induced by SARS-CoV2 and non-SARS-CoV2	Yin S, et al.	J Thromb Thrombolysis	x		x
Coagulation disorders in coronavirus infected patients: COVID-19 SARS-CoV-1 MERS-CoV and lessons from the past	Giannis D, et al.	Journal of Clinical Virology			
COVID-19-Related Severe Hypercoagulability in Patients Admitted to Intensive Care Unit for Acute Respiratory Failure	Spiezia L, et al.	Thromb Haemost. 2020	x		x
D-dimer levels on admission to predict in-hospital mortality in patients with Covid-19	Zhang L, et al.	J Thromb Haemost. 2020	x		
Epidemiological characteristics and clinical features of 32 critical and 67 noncritical cases of COVID-19 in Chengdu	Zheng Y, et al.	Journal of Clinical Virology	x		
Hematological findings and complications of COVID-19	Terpos E, et al.	Am J Hematol. 2020		x	
Hypercoagulability of COVID-19 patients in Intensive Care Unit. A Report of Thromboelastography Findings and other Parameters of Hemostasis	Panigada M, et al.	J Thromb Haemost. 2020	x	x	
Pathological evidence of pulmonary thrombotic phenomena in severe COVID-19	Dolhnikoff M, et al.	J Thromb Haemost. 2020		x	
The clinical data from 19 critically ill patients with coronavirus disease 2019: a single-centered observational study	Zhang J, et al.	Zeitschrift fur Gesundheitswissenschaften = Journal of public health	x		
Chinese expert consensus on diagnosis and treatment of coagulation dysfunction in COVID-19	Song JC, et al.	Military Medical Research		x	x
COVID-19 and hemostasis: a position paper from Italian Society on Thrombosis and Hemostasis (SISET)	Marietta M, et al.	Blood Transfusion			x
COVID-19 and Thrombotic or Thromboembolic Disease: Implications for Prevention, Antithrombotic Therapy, and Follow-up	Bikdeli B, et al.	Journal of the American College of Cardiology			x
Potential therapeutic effects of dipyridamole in the severely ill patients with COVID-19	Liu X, et al.	Acta Pharmaceutica Sinica B			x
RE: ISTH interim guidance to recognition and management of coagulopathy in COVID-19	Akima S, et al.	Journal of Thrombosis and Hemostasis	x		
The procoagulant pattern of patients with COVID-19 acute respiratory distress syndrome	Ranucci M, et al.	J Thromb Haemost	x		x
High incidence of venous thromboembolic events in anticoagulated severe COVID-19 patients	Llitjos JF, et al.	J Thromb Haemost		x	x
Incidence of thrombotic complications in critically ill ICU patients with COVID-19	Klok FA, et al.	Thromb Res		x	x
Practical diagnosis and treatment of suspected venous thromboembolism during COVID-19 Pandemic	Obi AT, et al.	J Vasc Surg Venous Lymphat Disord			x
Prevention and Treatment of Venous Thromboembolism Associated with Coronavirus Disease 2019 Infection: A Consensus Statement before Guidelines	Zhai Z, et al.	Thromb Hemostasis			x
Venous and arterial thromboembolic complications in COVID-19 patients admitted to an academic hospital in Milan, Italy	Lodigiani C, et al.	Thrombosis Research	x	x	x
Diagnosis, Prevention, and Treatment of Thromboembolic Complications in COVID-19: Report of the National Institute for Public Health of the Netherlands	Oudkerk M, et al.	Radiology	x		x

Papers selected after the two steps screening process. This table presents the content of the 39 studies.

### D-Dimer Values

D-dimer values (*n* = 25) (Table 3) were increased in all the studies, compared with general control population, in all COVID patients. D-dimer on admission greater than 4 times the upper limit of normal (ULN) could effectively predict in-hospital mortality in patients with COVID-19, which indicated D-dimer could be an early and helpful marker to improve management of COVID-19 patients.^13^ A mean increase in concentration is appraised in more severe patients, with higher concentrations suggesting a higher mortality rate.^13,24,31^ D-dimer levels higher than twice ULN are associated with worse outcome by 2 studies.^19,24^ The recommendation to hospitalize patients with values higher than 3-4 times the ULN was questioned by Akima et al.^44^ Patients with D- dimer higher than 6 times ULN seem to benefit the most from the use of heparin therapy.^48,49^

**TABLE 3:** D-Dimer and Complications

Authors (Study Size)	D-Dimer for Prognosis	Hypercoagulation or Hemorrhagic Manifestations
Li XY, et al.	Elevated - N/A in abstract	Highlighted the risk of VTE and PE
Zhang G, et al. (n=95)	For >1 μg/mL group: - 81.2% was severe cases - 71.9% reached ICU, mech vent or death	
Chen G, et al. (n=21)	5x normal	
Chen T, et al. (n=113)	Deceased patients mean: 4.6 μg/mL Recovered patients mean: 0.6 μg/mL	
Zhang JJ, et al. (n=140)	43% of patients had high D-dimer 60% of critical patients had more than 2x max D-dimer Nonsevere patients mean: 0.2 μg/mL (0.1 - 0.3) Severe patients mean: 0.4 μg/mL (0.2 - 2.4)	
Li X, et al. (n=25)	9/12 (75%) of patients had increased D-dimer	
Zhou F, et al. (n=191)	> 1 µg/L is associated with fatal outcome	
Wan S, et al. (n= 135)	Moderate patients mean: 0.3 (0.2 - 0.5) µg/L Survivor patients mean: 0.6 (0.4 - 1.1) µg/L	
Gao Y, et al. (n=43)	Mild group mean: 0.21 µg/L (IQR: 0.19 - 0.27) Severe group mean 0.49µg/L (IQR: 0.29 - 0.91)	
Ji HL, et al.		Evidence of microthrombi in the blood vessel
Cui S, et al. (n= 81)	D-dimer higher in DVT patients (mean 5.2 ± 3.0 µg/mL vs 0.8 ± 1.2 µg/mL) If 1.5 μg/mL was used as the cutoff value for D-dimer to predict DVT, the sensitivity, specificity, PPV* and NPV** were 85.0%, 88.5%, 70.8% and 94.7%, respectively D-dimer decrease after starting anticoagulant therapy	25% of all patients had DVT. No hemorrhagic complications
Han H, et al. (n=94)	Ordinary group mean: 2.14 ± 2.88 µg/mL Severe group mean: 19.11 ± 35.48 µg/mL Critical group mean: 20.04 ± 32.39 µg/mL	
Wu C, et al. (n=201)	No ARDS group: 0.52 (0.33 - 0.93) µg/mL ARDS group: 1.16 (0.46 to 5.37) µg/mL with ARDS: - survivors: 0.49 (0.31 to 1.18) µg/mL - deceased: 3.95 (1.15 to 10.96) µg/mL	
Lippi G, Favaloro EJ. (n=183)	5x normal with severe disease (Huang) 3.5x normal with severe disease (Tang) 2.5x normal with severe disease (Wang) Weighted mean: 2.97 µg/mL (95% CI: 2.47–3.46 µg/mL)	
Tang N, Li D, et al. (n= 183)	Survivor group: 0.61 (0.35-1.29) µg/mL Deceased group: 2.12 (0.77-5.27) µg/mL 85% non-survivors had D-dimer > 3 µg/mL	Most of the deaths (>75%) had thrombotic complications. No hemorrhagic complications
Tang N, Bai H, et al. (n= 449)	D-dimer >6x normal benefit from heparin therapy	
Yin S, et al. (n= 553)	Patients using heparin and with D-dimer > 6 x normal had significant lower mortality (32.8% vs. 52.4%)	
Spiezia L, et al. (n=22)	All ICU patients - 5,343 ± 2,099 μg/mL	
Zhang L, et al. (n= 343)	D-dimer ≥ 2.0 μg/mL on admission predict in-hospital mortality	
Zheng Y, et al. (n=99)	Mild group mean: 0.78 ± 0.76 µg/mL Severe group mean 2.65 ± 3.-3 µg/mL	
Panigada M, et al. (n=24)	ICU patients: 4,877 (1,197-16,954) µg/L	TEG results are compatible with a state of hypercoagulability, DVT and PE
Zhang J, et al. (n=19)	Survivor group: 0.48 (0.42-0.97) µg/mL Deceased group: 2.15 (1.4-9.2) µg/mL	
Akima S, et al.	3-4x normal should lead to hospital admission	
Ranucci M, et al. (n=16)	All ICU patients, given heparin 6-8000UI bid Baseline: 3.5 (2.5-6.5) µg/mL 7 days follow-up: 2.5 (1.6-2.8)	No hypercoagulation complications after starting therapy No hemorrhagic complications
Lodigiani C, et al. (n=388)	Survivors mean: ICU 1,5 µg/mL, ward 0.4 µg/mL Non-survivors mean: ICU 3,4 µg/mL, ward 0.9 µg/mL	7.7% globally had hypercoagulation events (16.7% in ICU, 6.6% in the wards). 60% of the events were PE 3 Strokes and 1 MI reported
Oudkerk M, et al.	2 - 4 µg/mL requires attention and accurate monitoring	

D-dimers data and hypercoagulation/hemorrhagic complications in the selected studies.

### Thrombotic and Hemorrhagic Complications

Manifestations of hypercoagulation (*n* = 6) (Table 3) are widespread, with the most common reported in reduced numbers: DVT, PE, ST segment myocardial infarction (STEMI), and cerebrovascular accident (CVA).^47^ No hemorrhagic complication was reported by any of the studies. Close monitoring is always suggested due to the increased risk of bleeding with anticoagulation following standard monitoring of symptoms, signs, and blood tests.

### Therapeutic Evidence

Except for Lin et al.,^2^ no study suggests a D-dimer cutoff to start anticoagulation therapy (*n* = 16) (Table 4). The criteria for beginning anticoagulation treatment with COVID-19 patients is debated, with some studies starting prophylactic anticoagulation on every hospitalized patient,^10,51^ others are based on the stratified risk of VTE,^52^ while some others suggest the inclusion of ambulatory patients with risk factors.^47^

**TABLE 4:** Therapeutic Approaches

Authors	Use of D-Dimer to Start Anticoag?	Initial Drug and Dose	D-Dimer Change to Escalate Anticoag.	Escalation Drug and Dose	DVT/PE Monitoring- Signs and Symptoms, Lab or Imaging	Anticoag. at Discharge
Cui S, et al.	w/o	No preventive anticoagulant was administered			US and CT	
Casini A, et al.	w/o	LMWH OR UFH “standard dose”, according to creatinine clearance and patient weight			Clinical monitoring	
Lin L, et al.	yes - 4 ULN	LMWH 100U/kg x 2/day				
Tang N, Bai H, et al.	w/o	Enoxaparin 40-60mg/day OR UFH 10-15K UI/day				
Yin S, et al.	w/o	Enoxaparin 40-60mg/day OR UFH 10-15K UI/day				
Song JC, et al.	w/o	1-2 doses of LMWH/day, until the patient’s DD level returns to normal	IF severe coagulation disfunction: lab to guide titration (TEG heparinase)			
Marietta M, et al.	w/o	LMWH 4K UI/day OR UFH OR Fondaparinux; if not possible use limb compression		LMWH OR UFH anticoagulant dose IF VTE/PE or other risk factors	US	Yes - up to 7-14 after discharge
Bikdeli B, et al.	w/o - according to risk stratification	LMWH OR UFH according to risk stratification			IF symptoms or high risk, US	Use of DOAC or LMWH in patient with increased DVT risk - some in the writing group also add D-Dimer >2 times reference
Liu X, et al.	w/o - group of selected patients	Dipyridamole 50mg				
Ranucci M, et al.	w/o - Started to all ICU patients	LMWH 4K UI/day	All escalated	LMWH 6K IU b.i.d. (or adjusted for body weight) + clopidrogrel if high PLT count		
Llitjos JF, et al.	w/o - Started to all ICU patients	Prophylactic and therapeutic anticoagulation			US	
Klok FA, et al.	w/o - Started to all ICU patients	Nandroparin prophylactic dosing				
Obi AT, et al.	w/o - all hospitalized patients	LMWH OR UFH prophylactic dosing	Wells score >4 with low bleeding risk and/or imaging for DVT/PE	LMWH therapeutic dose OR NOAC	US, CT for PE confirmation if high bleeding risk	1-2 month of anticoagulant, then imaging
Zhai Z, et al.	w/o - All critical patient, selected ambulatorial	LMWH OR UFH prophylactic dosing; mechanical compression additionally				Yes
Lodigiani C, et al.	w/o - all ICU patients	LMWH prophylactic dosing			Clinical monitoring	
Oudkerk O, et al.	w/o - all hospitalized patients	LMWH prophylactic dosing	If D-Dimer quickly increase and symptoms	LMWH full therapeutic dose	US, CT if needed	

Papers with data regarding the therapeutic approach.

Most studies suggest as a starting therapy LMWH, with dosing adjusted for weight and creatinine clearance (CCr) to be in prophylactic range or, alternatively, unfractionated heparin (UFH), depending on the CCr. The dosages would follow each institution’s standard dosage algorithms and are not discussed in this study after a careful standard assessment of bleeding risk and other contraindications. Just a single study suggests as first-line therapy the use of therapeutic doses of LMWH or UFH.^52^ Additionally, a study suggests using D-dimer OR thromboelastography (TEG) for monitoring therapeutic efficacy.^41^

While authors recognize the role of ultrasound (US) in the surveillance of DVT complications,^5,28,42^ no study suggests routine sonographic screening. The use of CTA is preferred by some authors in case of high bleeding risk,^10^ while clinical monitoring followed by US and CTA is still the most common approach,^10,51,52^ D-dimer^50^ is suggested only once in this findings as a tool to escalate therapy.

Escalation therapy is unclear in many studies. The studies that mention the increase of dosing, refer to the use of LMWH or UFH to full therapeutic dosing^42,50,52^ with just a single study suggesting the use of novel oral anticoagulant (NOAC).^10^

Few studies refer to the use of anticoagulant therapy after discharge, with direct oral anticoagulant (DOAC) or LMWH as reference drugs.^42,47,52^ Therapy duration is referred from 7 to 14 d^42^ up to 2 mo suggested by Obi et al.^10^

## LIMITATIONS

The highest level of evidence is a systematic literature review of randomized controlled studies with meta-analysis of data. When this study commenced at that phase of the COVID-19 clinical spectrum there were no such studies. Tricco et al^53^ raised the concern of poor quality of reporting of numerous rapid reviews and recommends a prospective study comparing the results of systematic literature reviews and scoping reviews as a measure of quality analysis of available data.

Unfortunately, at the time of this study, the incidence of thrombotic complications and outcomes of anticoagulation therapy are unknown due to lack of standardized reporting, lack of autopsy studies, and other data gaps. Most studies are retrospective and present small sample sizes. Additionally, the majority are focused on Chinese patients, which could lead to biases in population selection and therapeutic management.^12^ Studies are highly heterogeneous in reporting anticoagulant management and therapeutic suggestions, and not many papers have considered the possibility of escalating therapy to reduce the potential of thrombotic complications. Additionally, little evidence is available of the use of antiplatelet aggregation therapy, with a single study reporting its use,^43^ while most of the therapeutic approaches are based on anticoagulant use alone. This was not 1 of the search questions, but it is an open point for further clinical research.

Time zero has proven difficult to define when the initial D-dimer test was obtained in the time course of the COVID-19 spectrum in available studies. Each patient presents at a different point in time in their disease course, comparing patient to patient or an aggregate analysis of patients’ D-dimer levels would require a careful analysis.^54^ Perhaps in due time this can and likely will be defined, but in the midst of the evolving science of the COVID-19 spectrum, our only alternative is to appreciate the global phenomena of the hypercoagulable state and extrapolate what we do know in the absence of double-blind random control studies or a registry of studies that use the same treatment guideline with clinical outcome measurements, including autopsy, imaging, and other determinants of VTE and PE.

## DISCUSSION

### Pathophysiologic Effects of COVID-19

The binding of the SARS-CoV-2 virion to an angiotensin-converting enzyme 2 (ACE2) receptor, largely found in alveolar epithelial cells, activates cytokine release. In some individuals, the number of proinflammatory cytokines released sharply increases due to loss of negative feedback to the immune system, and positive feedback from the inflammatory cytokines leading to further production of inflammatory cytokines. This results in a phenomenon known as cytokine release syndrome (CRS), or cytokine storm, a dynamic process that may not be clinically apparent.

### CRS Lab Findings

Cytokine measurement is theoretically appealing, but most markers are not easily assessed in the peripheral blood and others have not yet proven to be predictive of poor outcomes.^55^ In 2002, Shorr et al.^56^ showed that D-dimer levels in critically ill patients correlate with activation of the proinflammatory cytokine cascade. Chen et al^20^ in a small sample size observation study of COVID-19 patients demonstrated similar with CRS markers and the D-dimer markedly (5 × ULN) elevated in the severe group (*n* = 11) than the moderate group (*n* = 10) with normal D-dimer levels. Neutrophil extracellular traps (NETs) are extracellular webs of chromatin, microbicidal proteins, and oxidant enzymes that are released by neutrophils to contain infections. However, when not properly regulated, NETs have potential to propagate inflammation and microvascular thrombosis. Zuo et al.^57^ found that patients with COVID-19 (*n* = 50 patients; *n* = 84 samples) have elevated levels of markers of NETs that strongly correlate with elevated D-dimer levels.

Importantly, these NET markers were higher in hospitalized patients receiving mechanical ventilation as compared with hospitalized patients breathing room air. Experience suggests that trends in laboratory studies rather than threshold values will be most informative.^58^ The D-dimer is readily available in most hospital laboratories and most providers are familiar with the association between VTE and elevated D-dimer levels. In a COVID-19 patient with CRS and worsening coagulopathy as evidenced by a rising D-dimer, it may make sense to administer escalated doses of anti-coagulation before thrombotic events occur.^39,58^

### TEG

TEG is a noninvasive test using blood obtained by means of venipuncture that can measure the ability of whole blood to form a clot. Over the years, TEG has been highly studied and used in various capacities, including assisting in liver transplant patients, cardiac surgery, and in traumatic blood loss. It can detect and quantify changes in the viscoelastic properties of a blood sample during clotting. Normal range for coagulation index (CI) is -3.0 to +3.0, where a CI > +3.0 would indicate a hypercoagulable state, and CI < -3.0 indicates a coagulopathy.^59^ A study conducted by Panigada et al.^38^ used TEG testing on 24 COVID-19 patients admitted to the ICU in Milan, Italy, to show evidence of hypercoagulability with a severe inflammatory state, but not acute DIC. Elevated TEG results were shown to correlate with elevated D-dimer results in COVID-19 patients in severe respiratory failure, supporting a hypercoagulable state.^60^ Chinese expert consensus supported the use of TEG in severe COVID- 19 patients in part because TEG was available and an actionable test, interpretable to assist management.^41^

### D-Dimer in CGs

D-dimer is a major fibrin degradation product released upon cleavage of cross-linked fibrin by plasmin. Although other fibrin degradation products are generated with this cleavage of fibrin, D-dimer is the most studied and validated for clinical assessment. Normal levels of D-dimer vary by laboratory, depending on their equipment and process. For the purpose of how to create a CG while interpreting observational studies from across the world, the D-dimer level will be expressed as “times ULN” or “× ULN”. Although not studied with COVID-19 patients using an assessment of pretest clinical probability with age-adjusted D-dimer, there was a low likelihood of clinical venous thromboembolism.^61^ The recommended (and most used) adjustment is by the formula for patients over 50 y of age is (patient’s age × 10) µg/L.^62^

The development of CGs to diagnose, treat, and monitor the hypercoagulable state with COVID-19 patients requires an appreciation of small sample observation studies. Although Lippi et al. established a weighted mean difference among 4 studies (Huang,^63^ Tang,^31^, Wang,^64^ and Zhou^24^) of a D-dimer level of 6 × ULN, acknowledging the heterogeneity across the studies, the main point raised is that the evolution of a worsening clinical course can be determined by serial D-dimer levels.

### Use of Direct Oral Anticoagulants

For over 50 y, warfarin was the only oral anticoagulant available. In the past 10 y, we have seen the development of NOACs. These drugs are now commonly classified as DOACs. The 5 DOACs currently available in the United States are dabigatran (Pradaxa®), rivaroxaban (Xarelto®), apixaban (Eliquis®), edoxaban (Savaysa®), and betrixaban (Bevyxxa®). DOACs are termed “direct” because they block a single blood-clotting factor to treat or prevent thrombus. These drugs have different and often overlapping FDA indications, but all indications are for the treatment or prevention of thromboembolic disease. Given the increased risk and prevalence of thrombotic complications in COVID-19, the question has been raised if DOACs would play a role in the treatment of or reduction of risk of VTE in this subset of patients.

Current consensus supports the universal administration of thromboprophylaxis for all patients admitted for COVID-19, who do not have an increased risk of bleeding or contraindication to anticoagulation.^3,12,46,65^ Many support a varied dosage strategy based on VTE risk (typically using D-dimer), weight, and renal function. Most experts recommend LMWH or fondaparinux over UFH, as it minimizes patient interaction with managing drips and eliminates difficulties maintaining therapeutic levels. It would seem likely that DOACs would offer these same advantages. However, most experts do not recommend DOACs for thromboprophylaxis or treatment of VTE for COVID-19 inpatients. The principal reason for this is due to interactions between investigational drugs for COVID-19 and DOACs. Multiple medications under investigation for COVID-19 have drug-drug interaction with DOACs due to their effect on the cytochrome-P450 enzyme activity, the enzyme-substrate by which most DOACs are metabolized. The University of Liverpool has collated a list of drug interactions at *http://COVID19-druginteractions.org/*.^66^ Given this, it is even recommended that patients on DOACs (and warfarin) should be switched to LMWH on admission for COVID-19, to dosages corresponding to DOAC or warfarin dosing.^67^

### Extended Thromboprophylaxis

The greatest use for DOACs to prevent VTE in the COVID-19 patient appears to be at discharge and in the outpatient treatment space. After hospital discharge from acute medical illness, extended prophylaxis with LMWH or DOAC’s can reduce the risk of VTE.^68,69^ While there are no current data specific to thromboprophylaxis in COVID-19, it stands to reason that, due to the increased risk of thrombotic events identified beyond what is typically seen in the acutely medically ill patient population, upon discharge COVID-19 inpatients could benefit from extended thromboprophylaxis. Many experts and academic institution guidelines support risk stratification for the use of DOACs for extended thromboprophylaxis postdischarge in patients without increased bleeding risk.^46,70^ Rivaroxaban, betrixaban, and apixaban have all demonstrated extended thromboprophylaxis in the acutely medically ill patients, in which patients with infectious diseases were part of their inclusion criteria^71-75^ (Supplementary Table 1, which is available online).^76,77^

These studies all compared the DOAC to enoxaparin, placebo, or enoxaparin plus placebo. The studies varied in patient population characteristics, inclusion criteria, and primary efficacy outcomes. Their results varied in superiority and noninferiority compared with the respective control group. Rivaroxaban and betrixaban did achieve FDA approval for the indication of extended thromboprophylaxis in the treatment of the acutely medically ill. There are no studies that have reviewed extended thromboprophylaxis in the treatment of COVID-19 patients. However, many experts, as well as the authors of this study, suggest constructing guidelines that strongly encourage the use of DOACs or LMWH for extended thromboprophylaxis postdischarge for all admitted COVID-19 patients without contraindications or increased risk of bleeding.^3,46,52,65^

With no studies in the COVID-19 thromboprophylaxis space, it is challenging to determine dosing at discharge for DOACs. Most experts prescribe dosages for DOACs that are typically in line with dosages studied in the thromboprophylaxis studies for treatment of the medically ill.

COVID-19 patients have an increased risk of thrombotic events, with many patients demonstrating markedly elevated D-dimer or have suspected or confirmed VTE and are treated as inpatients with enoxaparin at treatment doses. For those patients with confirmed VTE, the DOAC at discharge should be one that has an FDA approved indication for VTE treatment, and the dose should be the treatment regimen. For those patients who have markedly elevated D-dimer and treated with therapeutic doses of LMWH, confirming or ruling out VTE would be prudent before discharge. If there is no confirmatory VTE, one would expect that a COVID-19 patient who is well enough to be considered for discharge would have a D-dimer level that is at least below 3 times ULN and could be discharged on a DOAC or LMWH at prophylactic dosages.

As far as the duration of prophylactic treatment, expert opinion has ranged from 14 to 45 d.^3,47,52,65^ Again, given that clinicians are out of the realm of the randomized control trials in thromboprophylaxis of COVID-19 patients to create CGs, determining duration of treatment for thromboprophylaxis is challenging. How long inflammation and thrombotic derangements last after recovering from the COVID-19 acute lung injury spectrum remains unclear, because the incidence of post-COVID-19 acute lung injury spectrum VTE/PE and other thrombotic complications is unknown. Cohoon et al.^73^ as a subgroup analysis of the MAGELLAN study concluded that rivaroxaban for thromboprophylaxis among patients recently hospitalized for acute infectious diseases after 35 d reduced the occurrence of VTE. The risk/benefit ratio remains a clinical decision based on the in-patient team’s rationale for thromboprophylaxis to continue postdischarge. The authors of this study recommend consideration of up to a 35-d DOAC course with a careful assessment of continued risk, bleeding precautions, with close follow-up.

### DOAC’s at Emergency Department Discharge

Autopsy reports of COVID-19 patients have demonstrated PE, cerebrovascular (stroke) and coronary artery (myocardial infarction [MI]) emboli.^78-80^ It could be extrapolated that patients with milder pulmonary symptoms of COVID-19 could also benefit from thromboprophylaxis as outpatients with DOACs or LMWH. Some experts recommend that patients with mild and moderate pulmonary COVID-19 who have a high or moderate risk of VTE (using IMPROVEDD,^81^ among other validated scoring systems), began pharmacological thrombotic prevention,^46^ others believe pharmacological prophylaxis should be reserved for inpatients.^82^ This is a controversial topic currently without evidence-based recommendations. However, with the increase in thrombotic events, some consideration for thromboprophylaxis should be given to the COVID-19 patient at high risk for VTE with no other indications for admission.

Risk stratification using the D-dimer seems to be the best approach, because it has been shown to predict severity of illness and thromboembolic risk.^83^ ISTH guidance suggests any COVID-19 patient with a D-dimer great than 3 times ULN be admitted regardless of the presence of any other admission criteria.^12,78^ However, that still leaves the at-risk patient with a D-dimer less than 3 times ULN without guidance. Patients with VTE risk and D-dimer levels from 1 to 3 times ULN with no other criteria for admission still have significant risk, and perhaps guidance for prescribing DOACs for those without significant bleeding risk, at the same dosage and duration as those discharged inpatients, would be a life-saving maneuver.

### Aspirin Use

The hypercoagulable thrombotic potential for COVID-19 patients that do not require admission based on oxygen requirements or other supportive care does raise the question of aspirin therapy without standard contraindications. Guzik et al.^84^ summarize that COVID-19 may lead to plaque instability and myocardial infarction, which is a common cause of death in COVID-19 patients. The most common cardiac complications include dysrhythmia (atrial fibrillation, ventricular tachydysrhythmias, and ventricular fibrillation). A study from the Zhongnan Hospital of Wuhan University shows that 16 of the 138 COVID-19 patients required cardiologic intensive care after developing arrhythmias.^64^ In the Lombardy region of Italy during the study period in 2020, a total of 9806 cases of COVID-19 were reported in the study territory. During this period, 362 cases of out-of-hospital cardiac arrest (OHCA) were identified, compared with 229 cases identified during the same period in 2019 (a 58% increase). COVID-19 was diagnosed or suspected in 103 of the 362 OHCA patients.^85^ Studies presented earlier have demonstrated the occurrence of MIs and CVAs. For these reasons, the recommendation without standard contraindication is for 325 mg^86^ of aspirin daily for the extended thromboprophylaxis period stated earlier in the DOAC discussion of up to 35 d.

### COVID-19 VTE/PE Prophylaxis CG

**FIGURE 2:**
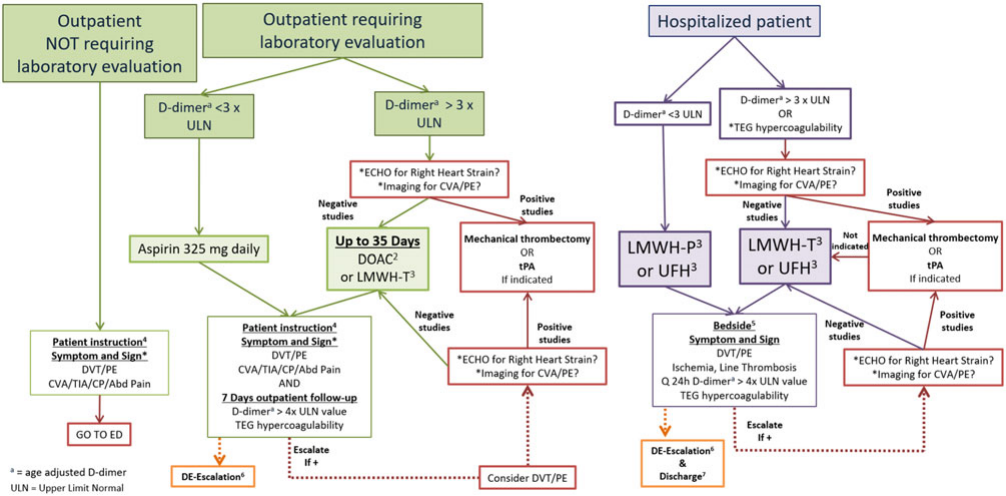
VTE/PE Thromboprophylaxis Clinical Guidelines for SARS-nCoV-2 PCR+/IgM+ (COVID+) or Person Under Investigation^1^

The CG developed (Figure 2 with accompanying Table 5) addressed the 4 main presentations of a COVID-19 patient: (1) not requiring further evaluation after confirmation based on the clinical exam, there is no recommendation to obtain a D-dimer at this time; (2) requiring further evaluation based on the clinical exam but not requiring hospitalization with a D-dimer that is normal or < 3 times ULN; (3) requiring hospitalization without risk or clinical indication for VTE or PE with a D-dimer that is normal or <3 times ULN; (4) requiring hospitalization with risk or clinical indication for VTE or PE or with a D-dimer > 3 times ULN. A key aspect of this CG is to educate the patient upon discharge from the initial presentation or after acute in-patient care to be aware of symptoms and signs of VTE: DVT, PE, chest pain or angina equivalents, CVA/transient ischemic attack (TIA), mesenteric ischemia, and limb ischemia from peripheral artery disease (PAD). If the patient has any concerns, they should be instructed to call 911 (or the local number for an ambulance) or go to the nearest emergency department.

**TABLE 5:** Key to Figure 2

**1.** **SARS-nCoV-2 PCR/IgM + (COVID +) or Person Under Investigation (PUI)** A. Past Medical History that would increase the risk of VTE ➢ Consider using Well’s Score,^ab^ PERC,^c^ IMPROVEDD,^d^ or other scoring system utilized per usual in institution Age > 65 years old^e^ ➢ History of bedridden, hemi-paralysis ➢ Body Mass Index (BMI): < 25, 25-35, >35 (each has different risk and adjust meds) ➢ Creatinine Clearance (CCr): < 30 > (each has different risk and adjusts meds) ➢ History of Heparin Induced Thrombocytopenia (HIT)/Heparin Induced Thrombocytopenia and Thrombosis (HITT) THEN use non-heparin alternative ➢ History of remote Venous Thromboembolism (VTE)/Pulmonary Embolism (PE) ➢ IF Major Surgery past 48 hours THEN ***Use mechanical prophylaxis per institution VTE prophylaxis protocol*** ➢ Thrombophilia: genetic or acquired (Factor V Leiden, Malignancy, Rheumatologic Disorders, etc.) B. CAUTION if there is an increased risk of bleeding: ➢ History of Atrial Fibrillation, Valve Replacement, Current VTE or PE treatment ➢ IF Maintenance Medication: Anti-coagulation, Anti-platelet THEN ***Consider switching patients taking direct oral anticoagulants (DOACs) or vitamin K antagonists (e.g. warfarin) to treatment dose LMWH when admitted*** ➢ IF Bleeding within past 48-72 hours THEN ***Use mechanical prophylaxis as above*** ➢ Liver Disease ➢ Lumbar Puncture/Epidural Catheter past 4 hrs or in the next 12 hours ➢ IF Major Surgery past 48 hours THEN ***Use mechanical prophylaxis as above*** C. Contraindication to anti-coagulation ➢ Alteplase (tissue Plasminogen Activator) use past 24 hours ➢ Baseline Prothrombin Time/International normalized ratio (PT/INR) > 2 ➢ Baseline Activated Partial Thromboplastin Time (aPTT) > 45 seconds ➢ Hemoglobin < 6 grams/deciliter (g/dl) ➢ Platelet Count < 50,000/microliter (μL) ** 2. DOAC (when not admitted or upon discharge)** ➢ Consider standard contraindications in addition caution with anti-viral therapy ➢ Dosing: ∘ Apixaban 2.5 mg by mouth twice daily ∘ Rivaroxaban 10 mg by mouth daily for 30 days ** 3. Monitor:** ➢ **LMWH: Low Molecular Weight Heparin; standard per institution precautions, dosing and** Anti-Xa assay peak level (drawn 3-5 hours after 3^rd^ dose) ➢ **UFH: Unfractionated Heparin; standard per institution precautions, dosing and maintenance** ➢ Change in SIC,^f^ ISTH DIC^g^ ➢ PT/INR, PTT, Fibrinogen (Mondays/Thursdays) ** 4. Discharge instructions per institution to go to the Emergency Department if:** ➢ VTE: one arm or leg swelling, pain, redness, warmth ➢ PE: chest pain, coughing (especially if coughing blood), increased heart rate, dyspnea ➢ Stroke/Transient Ischemic Attack ➢ Angina ➢ Abdominal Pain (mesenteric ischemia) ➢ Peripheral Artery Disease ** 5. Bedside** ➢ DVT/PE: if clinically indicated: Bilateral Lower Extremity US for DVT, ECHO for Right heart strain, TEG (Compare to prior if available) ➢ Thrombosis: Dialysis and ECMO catheter clot ➢ Clinical Sign end-organ ischemia: CVA/TIA, Chest pain or angina equivalent, Peripheral Artery disease, Abdominal pain consistent with mesenteric ischemia ➢ Q 24 D-dimer: in addition to routine labs, if ≥ 4 x ULN (age adjusted) ➢ Any positive THEN *** ESCALATE*** ** 6. De-Escalate: LWMH/UFH to DOAC or ASA per treatment team** ➢ Clinical improvement ➢ D-dimer improvement: in concert with clinical improvement, steady decreasing D-dimer/time to < ULN (age adjusted) ** 7. Discharge** ➢ Hospitalized Per Clinical parameters: per institution ➢ Outpatient Per Clinical parameters: per institution ➢ Standard Discharge instructions per institution for: DVT/PE; CVA/TIA/CP/Abd. Pain ➢ Close follow-up, recommend within 7 days: consider phone follow-up, telehealth, home health, office

a Well’s Criteria for DVT. https://www.mdcalc.com/wells-criteria-dvt. Accessed May 1, 2020.

b Well’s Criteria for Pulmonary Embolism. https://www.mdcalc.com/wells-criteria-pulmonary-embolism. Accessed May 1, 2020.

c PERC Rule for Pulmonary Embolism. https://www.mdcalc.com/perc-rule-pulmonary-embolism. Accessed May 1, 2020.

d Gibson C, Spyropoulos A, Cohen A, et al. The IMPROVEDD VTE Risk Score: incorporation of D-dimer into the IMPROVE score to improve venous thromboembolism risk stratification. *TH Open*. 2017;01(01):e56-e65. doi: 10.1055/s- 0037-1603929.

e People Who Are at Higher Risk for Severe Illness. CDC. https://www.cdc.gov/coronavirus/2019-ncov/need-extra-precautions/people-at-higher-risk.html). Accessed May 28, 2020.

f Sepsis-Induced Coagulopathy (SIC) Score. https://www.mdcalc.com/sepsis-induced-coagulopathy-sic-score. Accessed April 29, 2020.

g ISTH Criteria for Disseminated Intravascular Coagulation (DIC). https://www.mdcalc.com/isth-criteria-disseminated-intravascular-coagulation-dic#pearls-pitfalls. Accessed April 29, 2020.

The CG advocates for clinicians to be keen on these symptoms during the post-acute COVID-19 phase as the period of hypercoagulability has yet to be determined. While admitted, all providers, including nurses, should be hyperacute to any signs of thrombosis, of hemodialysis or other catheters, as well as during extracorporeal membrane oxygenation (ECMO). Rapid decrease in oxygenation or an increase in other parameters that indicate a PE should trigger an escalation in anticoagulation treatment before formal diagnosis. The decision to obtain initial and serial imaging for DVT or echocardiogram for right heart strain or other easily obtainable studies for PE is not easily determined by this scoping review and is left up to the individual treatment team. All treating providers and nurses need to be on the alert for any clinical symptoms or signs of end-organ ischemia as an indication of thrombosis: TIA/CVA, angina equivalent, mesenteric ischemia, limb ischemia, etc. The most effective process to develop a local CG is to initiate a Clinical Guideline Team of Scientists (Table 6) to study and maintain a focus on the evolving science.

**TABLE 6:** Clinical Guideline Team of Scientists

**Suggested Roster**	
Physicians	Critical Care, Emergency, Hematologist, Hospitalist, Primary Care
Nursing	Critical Care, Emergency, Primary Care, Ward
Hospital Services	Information Technology, Lab, Pharmacy
**Tasks**	
Data	Acquisition, Analysis
Literature Review	
Creation and Maintenance of Clinical Guidance	

## CONCLUSIONS

This study stands on the shoulders of all the clinical scientists who knew that their observations were important for other scientists to study and will continue to study. Clinicians have abrogated their role in the translational science process that uses evidence-based medicine in the development of the CG that they follow in their daily practice. In the midst of the COVID-19 pandemic, response clinicians have had to create their own CGs for many aspects of the COVID-19 clinical spectrum while they practice, acquiring information from their own institution, networks, and from available on-line publications ahead of print, in essence building the plane (CGs) as they fly (practice).

These CGs reflect the current available information obtained by a scoping review with the full expectation that refinements, particularly the use of the D-dimer as a hypercoagulable marker and the timing and dosages of LMWH, DOACs, and aspirin (and other medications) will reflect on-going study waiting for the randomized controlled trials and subsequent systematic literature reviews and meta-analysis. The cautious process outlined in the creation of these guidelines remains at the forefront to address the COVID-19 morbidity and mortality.

## Supplementary material

For supplementary material accompanying this paper visit http://dx.doi.org/10.1017/dmp.2020.195. click here to view supplementary material
