# Supplementary material for: How to Build the Plane While Flying: VTE/PE Thromboprophylaxis Clinical Guidelines for COVID-19 Patients
Source: Disaster Med Public Health Prep. 2020 Jun 16:1–15. doi: 10.1017/dmp.2020.195 (PMC7338398; doi:10.1017/dmp.2020.195)
Supplement: Supplementary file 1 [file S1935789320001950sup001.docx]

**Table 5 - review of DOAC studies in thromboprophylaxis in medically ill patients**

| DOAC | STUDY | METHOD | RESULTS | CONCLUSION | DOSE |
| --- | --- | --- | --- | --- | --- |
| Betrixaban*  Bevyxxa® | APEX  (Gibson 2017) | RCT in which patients hospitalized for acute medical illnesses received SQ enoxaparin (40mg daily) for 10+4 days plus oral placebo for 35 to 42days or SQ placebo for 10+4 days plus oral betrixaban (80mg once daily) for 35 to 42 days. An analysis was performed in three prespecified progressively inclusive cohorts;   1. Patients with elevated d-dimer 2. Patients with elevated d-dimer or age > 75 3. All enrolled patients   The primary efficacy outcome was a composite of asymptomatic proximal DVT and symptomatic VTE. The principal safety outcome was major bleeding**. | The primary efficacy outcome occurred in 8.5% of the enoxaparin + oral placebo group versus 6.9% if the betrixaban + SQ placebo group. Major bleeding** occurred in 0.6% of the enoxaparin + oral placebo group versus 0.7% of the betrixaban + SQ placebo group. | No significant difference was found between patients with an elevated d-dimer, but analysis provided evidence suggesting a benefit for betrixaban in the other two cohorts. | 80mg po once daily |
| Rivaroxaban*  Xarelto® | MARINER  (Raskob  2016) | RCT that looked at 10mg rivaroxaban dose give to hospitalized patients at discharge with an IMPROVE Score of 4 or higher or a score 2 to 3 plus a d-dimer level of more the twice normal versus placebo for 45 days. Primary efficacy outcome was a composite of symptomatic VTE or death due to VTE. Principal safety outcome was major bleeding**. | Of the 12,019 in the intention-to treat analysis a primary efficacy outcome occurred in 0.83% of the rivaroxaban patients versus 1.10% of the placebo group. Major bleeding** occurred in 0.28% in the rivaroxaban group and 0.15% in the placebo group, | Rivaroxaban given to the described medically ill patients after hospital discharge was not associated with a significant lower risk of symptomatic VTE and death due to VTE than placebo. The incidence of major bleeding** in the rivaroxaban group was low. | 10 mg po once daily |
|  | MAGELLAN  (Cohen 2013) | RCT that studied patients 40 years of age or older who were hospitalized for acute medical illness to receive either SQ enoxaparin 40mg qd for 10+4 and oral placebo for 35+4 days or SQ placebo for 10+4 and oral rivaroxaban for 35+4 days. Primary efficacy outcomes were the composite of asymptomatic or symptomatic VTE up to day 10 (non-inferiority test) and up to day 35 (superiority test). Principal safety outcome was major or clinically relevant major bleeding**. | A primary efficacy outcome event occurred in 2.7% of the rivaroxaban + SQ placebo group and 2.7% in the enoxaparin + PO placebo group at day 10. A primary efficacy outcome occurred in 4.4% of the rivaroxaban + SQ placebo group and 5.7% in the enoxaparin + PO placebo group at day 35. A principal safety outcome event occurred in 2.8% of the rivaroxaban + SQ placebo group and 1.2% in the enoxaparin + PO placebo group at day 10 and 4.1% and 1.7% in the respective groups at day 35. | Rivaroxaban was found to be noninferior to enoxaparin for standard duration thromboprophylaxis (day 10), and that extended duration reduced the risk of VTE compared to enoxaparin plus placebo. Rivaroxaban was associated with increased risk of major bleeding**. A retrospective analysis was conducted of MAGELLAN and a subgroup consisting of patients with following findings were isolated.   - Pulmonary bronchiectasis, pulmonary hemorrhage or cavitation - Active cancer undergoing treatment - Active duodenal ulcer in last 3 months prior to treatment - Active bleeding** in the last 3 months prior to treatment - Receiving dual anti-platelet therapy   After removal of the subgroup from the Magellan cohort, rivaroxaban was found to have a more favorable risk benefit profile and no significant increase in major bleeding** compared to control group. | 10mg po once daily |
| Apixaban  Eliquis® | ADOPT  (Goldhaber 2011) | RCT in which acutely ill patients with CHF or respiratory failure or other medical disorders and at least one additional risk factor of VTE and who were hospitalized with an expected stay of at least three days to receive apixaban, (2.5mg bid) for 30 days versus enoxaparin SQ 40mg once daily for 6 to 14 days. The primary efficacy outcome was the 30-day composite of death related to VTE, PE, symptomatic DVT, or asymptomatic proximal-leg DVT as detected by USG on day 30. The primary safety outcome was bleeding**. | Among the evaluated patients, 2.71% met the principal efficacy outcome in the apixaban group and 3.06% in the enoxaparin group. By day 30, major bleeding** had occurred in 0.47% in the apixaban group and 0.19% in the enoxaparin group. | In medically ill patients, an extended course of thromboprophylaxis with apixaban was not superior to a shorter course with enoxaparin. Apixaban was associated with more major bleeding** events than was enoxaparin. | 2.5mg po bid |

*FDA approved for treatment of medically ill patients

** Definition of major bleeding varied in studies
